# Natural Occurrence of Regulated and Emerging Mycotoxins in Wheat Grains and Assessment of the Risks from Dietary Mycotoxins Exposure in China

**DOI:** 10.3390/toxins15060389

**Published:** 2023-06-09

**Authors:** Xiaofeng Ji, Canghong Jin, Yingping Xiao, Meihua Deng, Wen Wang, Wentao Lyu, Jiapeng Chen, Rui Li, Yan Li, Hua Yang

**Affiliations:** 1 State Key Laboratory for Managing Biotic and Chemical Threats to the Quality and Safety of Agro-Products, Institute of Agro-Product Safety and Nutrition, Zhejiang Academy of Agricultural Sciences, Hangzhou 310021, China; jixiaofeng@zaas.ac.cn (X.J.); ypxiaozaas@hotmail.com (Y.X.); meihuad@163.com (M.D.); wangwen@zaas.ac.cn (W.W.); lvwt@zaas.ac.cn (W.L.); microvet@163.com (R.L.); 2 School of Computer and Computing Science, Hangzhou City University, Hangzhou 310015, China; jinch@hzcu.edu.cn (C.J.); jiapengch@163.com (J.C.); 3 Department of Veterinary Medicine, Institute of Preventive Veterinary Sciences, College of Animal Sciences, Zhejiang University, Hangzhou 310058, China; yanli3@zju.edu.cn

**Keywords:** wheat grains, regulated mycotoxins, emerging mycotoxins, dietary exposure

## Abstract

Wheat grains are susceptible to contamination with various natural mycotoxins including regulated and emerging mycotoxins. This study surveyed the natural presence of regulated mycotoxins, such as deoxynivalenol (DON) and zearalenone (ZEN), and emerging mycotoxins such as beauvericin (BEA), enniatins (ENNs such as ENA, ENA1, ENB, ENB1) and Alternaria mycotoxins (i.e., alternariol monomethyl ether (AME), alternariol (AOH), tenuazonic acid (TeA), tentoxin (TEN), and altenuene (ALT)) in wheat grains randomly collected from eight provinces across China in 2021. The results revealed that each wheat grain sample was detected with at least one type of mycotoxin. The detection rates of these mycotoxins ranged from 7.1% to 100%, with the average occurrence level ranging from 1.11 to 921.8 µg/kg. DON and TeA were the predominant mycotoxins with respect to both prevalence and concentration. Approximately 99.7% of samples were found to contain more than one toxin, and the co-occurrence of ten toxins (DON + ZEN + ENA + ENA1 + ENB + ENB1 + AME + AOH + TeA + TEN) was the most frequently detected combination. The dietary exposure to different mycotoxins among Chinese consumers aged 4–70 years was as follows: 0.592–0.992 µg/kg b.w./day for DON, 0.007–0.012 µg/kg b.w./day for ZEN, 0.0003–0.007 µg/kg b.w./day for BEA and ENNs, 0.223–0.373 µg/kg b.w./day for TeA, and 0.025–0.041 µg/kg b.w./day for TEN, which were lower than the health-based guidance values for each mycotoxin, with the corresponding hazard quotient (HQ) being far lower than 1, implying a tolerable health risk for Chinese consumers. However, the estimated dietary exposure to AME and AOH was in the range of 0.003–0.007 µg/kg b.w./day, exceeding the Threshold of Toxicological Concern (TTC) value of 0.0025 µg/kg b.w./day, demonstrating potential dietary risks for Chinese consumers. Therefore, developing practical control and management strategies is essential for controlling mycotoxins contamination in the agricultural systems, thereby ensuring public health.

## 1. Introduction

Mycotoxins are secondary fungal metabolites synthesized by various fungal species, primarily those belonging to the genera Aspergillus, Penicillium, Fusarium, Alternaria, and Claviceps, under favorable environmental conditions [1]. These secondary metabolites are toxic and cause adverse impacts on human health, economic losses, and trade barriers [2]. The most commonly observed mycotoxins that present a concern to human health and livestock include aflatoxins (AFs), Ochratoxin A (OTA), Fusarium toxins (i.e., deoxynivalenol (DON), zearalenone (ZEN), fumonisins (FB_1_, FB_2_, FB_3_), HT-2 and T-2 toxins) [3], which are strictly regulated by many countries and international organizations worldwide [4]. Recently, considerable attention has been paid to a group of mycotoxins called emerging mycotoxins with limited toxicity and toxicokinetic data in vivo, namely Fusarium mycotoxins (i.e., beauvericin (BEA), enniatins (ENNs such as ENA, ENA1, ENB, ENB1)) and Alternaria mycotoxins (i.e., alternariol monomethyl ether (AME), alternariol (AOH), tenuazonic acid (TeA), tentoxin (TEN), and altenuene (ALT)) due to their frequent detection in various food commodities [4]. Some fungi can produce more than one mycotoxin. Moreover, more than one mycotoxin can be detected in a single food matrix [5]. Therefore, investigating natural mycotoxin contamination in agricultural products is essential and deserves attention to ensure access to safe and healthy food for human beings and to reduce the burden of foodborne diseases.

Wheat, a crop that develops together with human civilization, is one of the most important sources of carbohydrates in daily life globally. Humans commonly consume various wheat products, such as breakfast steamed buns, sandwiches, pasta, dumplings, pizza, or tea snacks of biscuits, puffs, and doughnuts, on a daily basis. However, wheat grains are commonly contaminated with natural mycotoxins resulting from various toxigenic fungi following pre- and post-harvest [3]. As mycotoxins are relatively stable during food processing, these natural mycotoxins might be transferred into the food production chain. The most common mycotoxins associated with wheat grains are DON, nivalenol (NIV), ZEN, ergotamine (Ergot), OTA, aflatoxins (Afla), and citrinin (CIT). Among these, DON and ZEN are two predominant mycotoxins, which have been frequently reported to contaminate wheat grains worldwide [6,7]. In China, the greatest mean prevalence of DON and ZEN was reported to be 17,753 μg/kg [8] and 275.9 μg/kg, respectively. High levels of BEA and ENA1 contamination were reported in Morocco, with 2000 μg/kg for BEA and 209,000 μg/kg in wheat grains, respectively [9]. To date, only a few studies have reported the occurrence of emerging Alternaria toxins [10,11], and limited studies have reported the prevalence of emerging BEN and ENNs in wheat grains [12]. Subsequent risk assessments of emerging mycotoxins for consumers are also scanty.

Mycotoxins can be detected by multiple analysis methods including ordinary test methods, such as high-performance liquid chromatography (HPLC) and liquid chromatography-mass spectrometry (LC–MS), rapid detection methods such as enzyme-linked immunoassay and immunochromatography, and other more advanced detection methods such as biosensor detection, nucleic acid aptamer fluorescence assay, optical waveguide pattern spectrum detection, and molecular imprinted polymer analysis [13,14]. Due to low detection and quantification limits, high efficiency, and accuracy, LC–MS/MS has become the most widely used technique for detecting mycotoxins in food commodities [14]. Considering different characteristics of multi-mycotoxins in wheat grains and a large number of daily inspection tasks, developing a simple sample pretreatment method with accurate quantitation is essential.

Although some studies have determined the occurrence of natural mycotoxins in wheat grains, only a few have paid attention to emerging mycotoxins. In addition, the occurrence patterns of mycotoxins contamination in wheat grains vary worldwide, within large countries or across years due to the influence of meteorological factors such as temperature, relative humidity, and precipitation. Continuous follow-up evaluation of natural mycotoxins contamination in wheat grains is the most effective way to ensure consumers’ access to safe and healthy food worldwide. To the best of our knowledge, dietary risk assessments associated with regulated and emerging mycotoxins in wheat grains in China are currently lacking. In this context, the present study aims to develop a suitable analysis method to determine the major regulated and emerging mycotoxins in wheat grains. The occurrence of multi-mycotoxins in wheat grains randomly collected from the main wheat producing areas across China was investigated and dietary exposure to these toxins among Chinese consumers was estimated.

## 2. Results and Discussion

### 2.1. Method Validation

The developed method was validated for linearity, accuracy (recoveries), precision (RSDs), the limit of detection (LOD), and the limit of quantification (LOQ) under guidance from SANCO/12495/2011 [15]. Recoveries of analyzed mycotoxins in the wheat grain samples were evaluated by spiking the samples with standards at three different concentrations (*n* = 6). In the national standard of People’s Republic of China GB 2761-2017 [16], the maximum limits for DON and ZEN in wheat grains are 1000 μg/kg and 60 μg/kg, respectively. Recoveries for DON were determined at 100, 1000, and 2000 μg/kg fortification levels, whereas those for ZEN were determined at 20, 50, and 100 μg/kg fortification levels. Due to the lack of maximum limits for emerging mycotoxins, low, medium and high fortification levels (in the range of 1 to 100 μg/kg) were applied for the recovery tests for emerging mycotoxins analyzed in this study (Table 1). As shown in Table 1, the average recoveries were in the range of 87.9–112%, while the relative standard deviations (RSD%) were all <11.9%. The LODs and LOQs were defined based on the signal-to-noise ratio (S/N) of 3:1 and 10:1, respectively, with the lowest matrix standard concentration under guidance from SANCO/12495/2011 [15]. As shown in Table 2, the LODs ranged from 0.03 to 3.0 µg/kg, and the LOQs ranged from 0.1 to 10.0 µg/kg in wheat grain samples. The results of method validation indicated the feasibility of the developed method. The method validation results verified the feasibility of the developed method for the determination of regulated and emerging mycotoxins in wheat grains. Figure S1 shows the LC–MS/MS chromatogram of mycotoxins DON, TeA, BEA, ENA, ENA1, ENB, ENB1, AME, AOH, TeA, TEN, and ALT in the wheat grain matrix.

### 2.2. Natural Occurrence of Mycotoxins in Wheat Grains

The detection frequency and concentration of the twelve mycotoxins in the wheat grain samples collected from China are presented in Table 3. A total of three hundred twenty-one wheat grain samples from eight provinces in China collected in 2021 were analyzed for two regulated mycotoxins and ten emerging mycotoxins. Overall, each wheat grain sample was detected with at least one type of mycotoxin. The detection rates of these mycotoxins ranged from 7.17 to 100%, with the average occurrence level ranging from 1.11 to 921.8 µg/kg. DON and TeA were the predominant contaminants with respect to both prevalence and concentration.

#### 2.2.1. Regulated Mycotoxins

DON and ZEN belong to type B trichothecene mycotoxins produced by several Fusarium fungi that commonly infect cereal grains [14]. DON exposure may lead to the occurrence of acute gastrointestinal diseases. ZEN has been associated with estrogenic syndromes [18]. To safeguard human health, the maximum levels of DON and ZEN in cereal grains and cereal grain-based products have been set by different countries and international organizations owing to their potential toxicity. In China, the maximum limit of DON and ZEN in wheat grains is 1000 μg/kg and 60 μg/kg, respectively [16]. The Codex Alimentarius Commission (CAC) set legislation on the maximum permitted limit only for DON in wheats (2000 μg/kg). The European Commission (EC 1881/2006) has established legislation on DON (1250 μg/kg) and ZEN (100 μg/kg) in wheat grains. In this study, the data on acylated derivative of DON are not provided due to its relatively low occurrence levels and detection frequencies compared with those of DON.

DON is a predominant contaminant of cereals and cereal-based food products worldwide [19]. As shown in Table 3, 95.6% (307/321) of wheat grain samples were found to be contaminated with DON at levels ranging from 3.50 to 8116 µg/kg, with a mean of 921.8 µg/kg. Of the total samples, 68.8% showed DON levels within the LOQ–1000 µg/kg, 20.2% within 1000–2000 µg/kg, 10.6% within 2000–3000 µg/kg, and 0.312% more than 3000 µg/kg (Table 4). Among the DON samples, 31.2% (100/321) exhibited the DON contamination levels exceeding the maximum tolerance limit of 1000 µg/kg, as stipulated by the Chinese government [15], and the highest level of DON exceeded the Chinese regulation by nearly eight and one-tenth-fold. According to previous studies in China, DON contamination in wheat grains varies across years and different sampling areas [20,21,22,23]. The average levels of DON (2706.3 µg/kg) were reported to be higher than that in our study by Chen et al., (2020) [20]. Moreover, a relatively low average level of DON than that of our study has been reported in previous studies, with concentration levels ranging between 82.1 and 500 µg/kg [18,20].

Wheat grains are susceptible to contamination with ZEN owing to the ubiquitous nature of Fusarium spores [24]. In this study, ZEN was detected in 53.6% (173/321) of analyzed samples, with a mean level of 19.7 µg/kg and a highest level of 220 µg/kg. As shown in Table 4, 96.6% of samples were contaminated with ZEN levels within the LOQ–60 µg/kg, 0.312% within 60–100 µg/kg, 2.49% within 100–200 µg/kg, and 0.623% more than 200 µg/kg. The highest level of ZEN exceeded the Chinese regulation by nearly three and six-tenths-fold. Among the wheat grains detected with ZEN, 3.4% (11/321) of samples were with occurrence levels in the range of 66.4–220.1 µg/kg, exceeding the Chinese regulation of 60 µg/kg [17]. According to the statistical analysis cited in the literature, the highest and lowest ZEN contamination levels in wheat grains have been reported in China (22572 µg/kg) and India (11.5 µg/kg), respectively [20,25]. Owing to their stability throughout the industrial chain production and processing, DON and ZEN contamination in wheat grains should not be ignored.

#### 2.2.2. Emerging Mycotoxins

The emerging mycotoxins BEA and ENNs (i.e., ENA, ENA1, ENB, and ENB1) are cyclic depsipeptides produced by a wide variety of Fusarium fungi. In this study, the detection frequency of BEA was 11.8%, with a mean concentration of 2.44 µg/kg and a highest level of 27.0 µg/kg. The observed rates are lower than the quantities of BEA previously reported in wheat grains [9,26,27] especially that of BEA (up to 4000 µg/kg) detected in wheat grains harvested from Morocco [9]. In the present study, ENNs were detected in 33.6–75.7% of samples, with an occurrence level of 0.20–190 µg/kg in samples. Zinedine, Fernandez-Franzon, Manes, and Manyes (2017) reported astonishingly high levels of ENB1 (795,000 µg/kg) in wheat [28]. Previously, the level of ENNs detected in wheat grains from Morocco was reported to be 209 mg/kg [9] and that detected in the samples from Tunisia was 180.6 mg/kg [24]. Furthermore, Juan et al., (2013) reported lower level of ENNs of 0.2–190 µg/kg [26]. Although no recommended levels of BEA and ENNs have been set, their prevalence in wheat grains might have adverse effects on human health, especially on infants and elderly people, which is a serious concern.

In recent years, the emerging Alternaria toxins have become a concern due to their frequent detection in wheat grains [10,11,29]. In the present study, the detection rate of Alternaria toxins from the order of high to low was as follows: TeA (100%) > TEN (99.4%) > AME (67.0%) > AOH (62.3%) > ALT (7.17%), whereas the average contamination level of Alternaria toxins from the order of high to low was as follows: TeA (331.9 µg/kg) > TEN (37.0 µg/kg) > AOH (8.72 µg/kg) > AME (4.89 µg/kg) > ALT (2.05 µg/kg). Among Alternaria toxins, TeA had the highest detection rate (up to 100%) and the highest contamination level (up to 2034 µg/kg), which are in line with those reported previously [10,11,26]. As shown in Table 4, 33.0% of samples were contaminated with TeA levels within the LOQ–500 µg/kg, 51.7% were contaminated within 200–500 µg/kg, 11.8% within 500–1000 µg/kg, 3.12% within 1000–2000 µg/kg, and 0.312% were contaminated with TeA levels > 2000 µg/kg. The high frequency and high content of TeA in the wheat grains may be attributed to the toxigenic capacity of Alternaria strains colonizing the wheat [10]. In this study, the average occurrence levels of AME and AOH were relatively lower than those reported previously [10,11,29]. Due to the mutagenicity and carcinogenicity of AME and AOH and their relevance to the etiology of human esophageal cancer [30], attention must be paid to the presence of AME and AOH in wheat grains from the human health perspective.

Compared with AME, AOH, and TeA, TEN and ALT have been relatively less studied in wheat grains and wheat-based products [31]. In this study, the detection rate of TEN in wheat grains was 99.4%, which was significantly higher than that of ALT (7.17%). The occurrence levels of both TEN (1.06–133 µg/kg) and ALT (1.0–6.17 µg/kg) were low in wheat grain samples. The highest TEN concentrations were reported in legumes and oilseeds [21]. On the other hand, relatively higher occurrence levels of TEN and ALT were reported previously. Muller and Korn (2013) reported that 2.6% (7/267) of wheat samples were contaminated with ALT (61.6–196.6 µg/kg) [32]. Xu et al., (2016) reported TEN occurrence levels to be in the range of 0.4–258.6 µg/kg, with a detection rate of 77% (285/3710) and an average occurrence rate of 43.8 µg/kg [11]. Most studies on the wheat grain samples have not focused on TEN and ALT; thus, limited information is available for comparison with the present results.

### 2.3. Geographical Distribution of Mycotoxins in Wheat Grains

**Figure 1:**
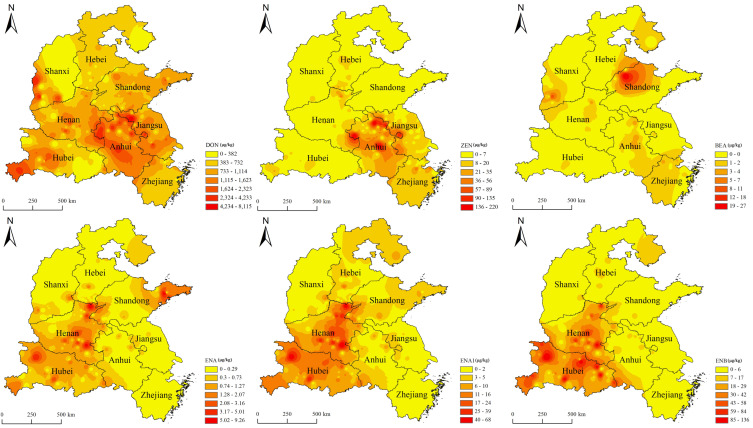

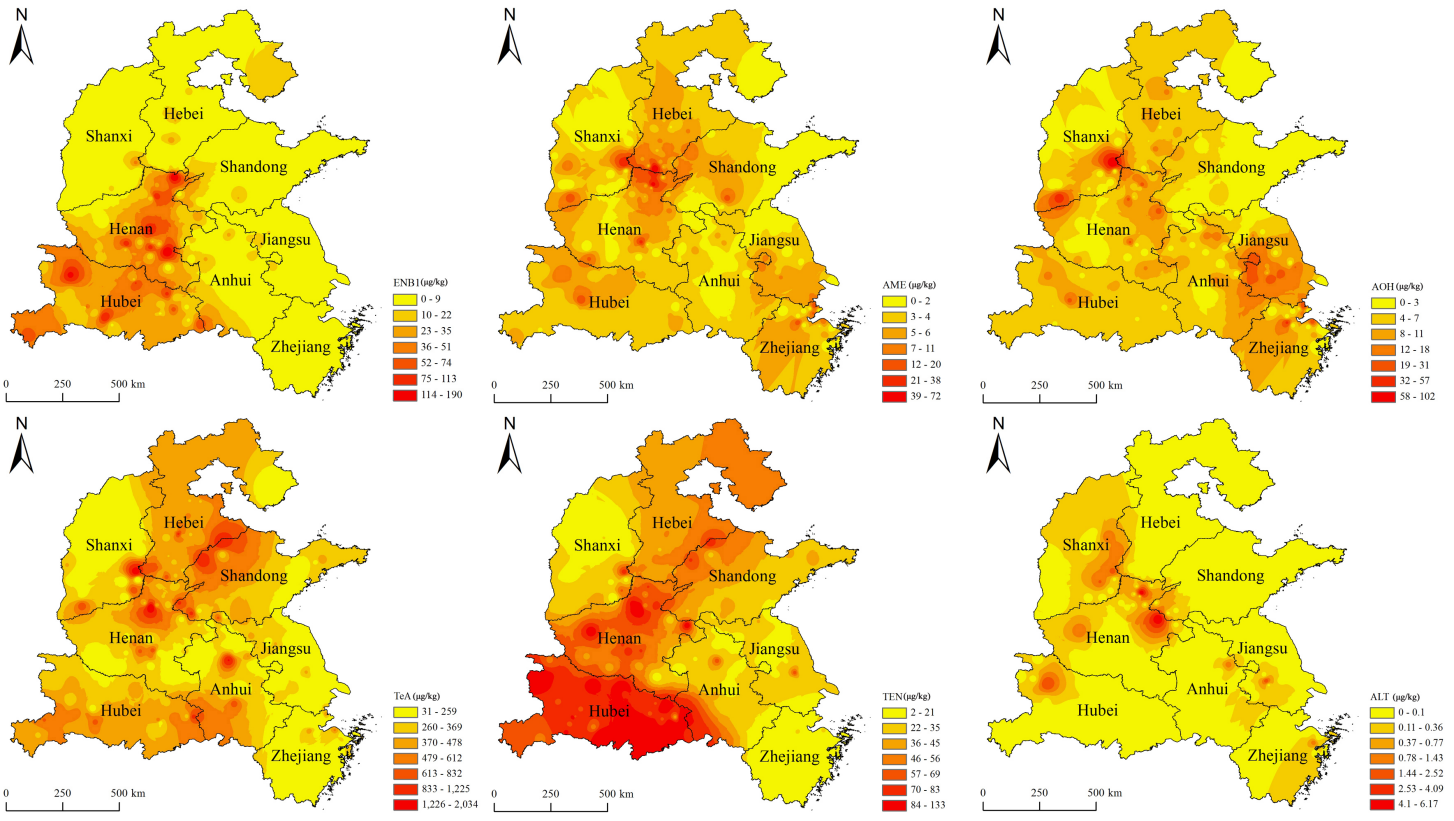
The spatial distribution of mycotoxins (i.e., DON, ZEN, BEA, ENA, ENA1, ENB, ENB1, AME, AOH, TEN, TeA, and ALT) in wheat grain samples collected across China in 2021.

The investigation outcomes of mycotoxins in wheat grains harvested across eight provinces in China are summarized in Table 5. Figure 1 depicts the spatial distribution of mycotoxins in wheat grain samples collected across China in 2021.

In this study, the wheat grain samples were collected from eight major wheat-producing areas in China, including four provinces in the north of the Huaihe River (e.g., Zhejaing, Jiangsu, Anhui, and Hubei provinces) and four provinces in the south of Huaihe River (e.g., Henan, Shandong, Shanxi, and Hebei). Huaihe River acts as the climatic boundary between the southern and northern regions of China, with the northern region of the river being a warm temperate zone and the southern region of the river being a subtropical zone [33]. The level of contamination of wheat samples with mycotoxins has been observed to vary across different geographies in China. Regarding the detection rates of mycotoxins in wheat grains, the Fusarium toxins (i.e., DON, ZEN, BEA, ENA, ENA1, ENB, ENB1) were detected in the order from high to low as follows: Hubei (50.8%) > Henan (50.7%) > Hebei (47.5%) > Anhui > (46.3%) > Jiangsu (40.0%) > Zhejiang (34.8%) > Shandong (32.2%) > Shanxi (21.3%), while the Alternaria toxins (AME, AOH, TEN, TeA, and ALT) were detected in the order from high to low were as follows: Hubei (80.0%) > Hebei (76.3%) > Henan (75.3%) > Jiangsu (70.2%) > Shandong (67.8%) > Anhui (64.9%) > Zhejiang (58.5%) > Shanxi (53.6%).

According to the spatial analysis, Anhui showed the highest average of DON (1649 µg/kg) and ZEN (59.7 µg/kg), Shandong demonstrated the highest average of BEA (13.9 µg/kg) and TeA (537µg/kg), Henan showed the highest average of ENNs (23.6 µg/kg), Henan demonstrated the highest average of AME (7.18 µg/kg) and ALT (3.73 µg/kg), Hubei showed the highest average of TEN (80.1 µg/kg), and Shanxi demonstrated the highest average of AOH (25.9 µg/kg). A study reported that the top four regions for DON contamination in wheat grains are located mainly in southern China (e.g., Anhui, Zhejiang, and Jiangsu), which is consistent with our findings [34]. The present study results provide new insights into the geographic profile of mycotoxins in wheat grains, especially for emerging toxins, suggesting that special attention must be paid to the contamination of wheat grain with certain toxins in different provinces in China and an effective management strategy must be implemented for controlling mycotoxin production.

### 2.4. Co-Occurrence of Mycotoxins in Wheat Grains

**Figure 2:**
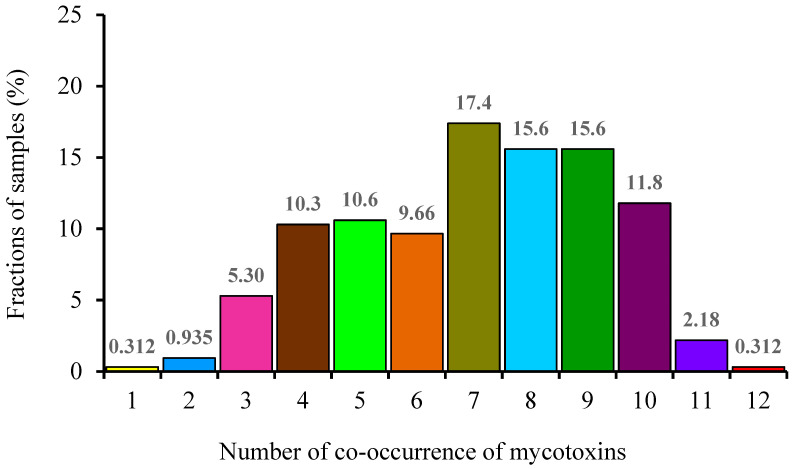
The frequency of co-occurrence of mycotoxins in wheat grain samples.

The co-occurrence of mycotoxins in wheat grains was statistically analyzed in this study. Figure 2 shows the frequency of co-occurrence of mycotoxins in wheat grain samples. The co-occurrence of more than one toxin was detected in 99.7% (320/321) samples. The co-occurrence of seven toxins accounted for the largest proportion (17.4%), followed by that of eight and nine toxins (15.6%), ten toxins (11.8%), five toxins (10.6%), four toxins (10.3%), six toxins (9.66%), three toxins (5.30%), eleven toxins (2.18%), two toxins (0.935%), one toxin (0.312%), and twelve toxins (0.312%). Table 6 summarizes the combinations of co-occurrence of regulated and emerging mycotoxins detected in the wheat grain samples. The top three combinations showing co-occurrence of a large number of mycotoxins were as follows: co-occurrence of ten toxins (DON + ZEN + ENA + ENA1 + ENB + ENB1 + AME + AOH + TeA + TEN), co-occurrence of nine toxins (DON + ENA + ENA1 + ENB + ENB1 + AME + AOH + TeA + TEN), and co-occurrence of eight toxins (DON + ZEN + ENB + ENB1 + AME + AOH + TeA + TEN). Blesa, Moltó, Akhdari, Mañes, and Zinedine (2014) reported that 51% of the wheat grains from Morocco were contaminated with two to six mycotoxins. The most frequent co-occurrence was noted with the combination ENA + ENA1 + ENB + ENB1 [35]. Juan et al., (2013) reported that 81% of cereal samples were contaminated with more than one mycotoxin in Italy [26].

**Figure 3:**
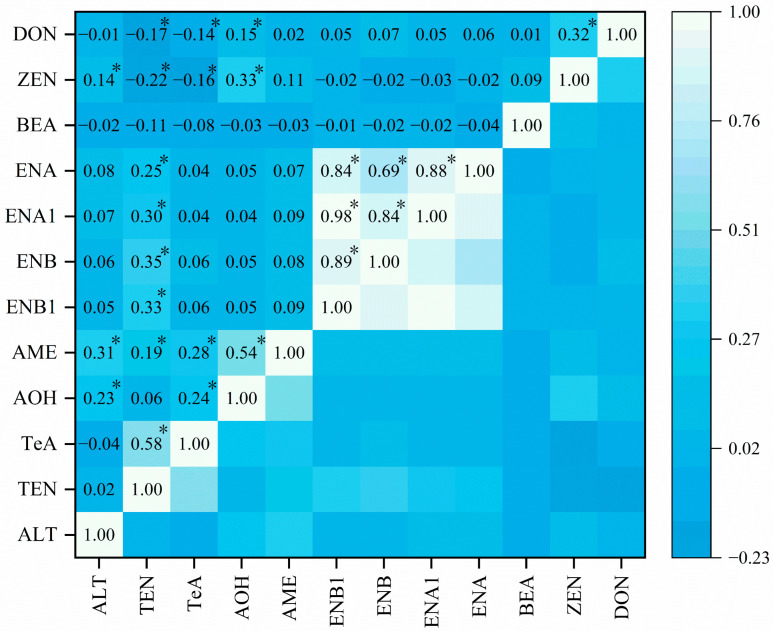
Correlation analysis among mycotoxins detected in wheat grain samples. * represents the statistically significant level (*p* < 0.05).

Correlation analysis was performed to determine the correlation among the mycotoxins detected in wheat grain samples (Figure 3). DON and ZEN (*r* = 0.32, *p* < 0.05), AME and AOH (*r* = 0.54, *p* < 0.05), TeA and TEN (*r* = 0.58, *p* < 0.05), among ENNs (*r* = 0.69–0.98, *p* < 0.05) demonstrated significant linear regressions of correlation, which are in line with the results of previous studies [8,36]. The co-occurrence of mycotoxins is a serious concern to human health owing to the possible synergistic and additive effects induced by multi-mycotoxins [37,38]. Exploring the co-occurrence of mycotoxins in wheat grains can provide comprehensive information for human health risk assessment.

### 2.5. Risk Assessment

A deterministic approach was applied to estimate the dietary risk of natural mycotoxins detected in wheat grains to Chinese consumers aged 4–70 years. The mean concentrations of DON, ZEN, BEN, ENNs, AME, AOH, TeA, TEN, and ALT in the wheat grain samples were 881.7, 10.6, 0.377, 5.85, 3.33, 5.98, 331.9, 36.8, and 0.286 μg/kg, respectively. Regarding the loss of mycotoxins during the process of wheat to wheat-based products, the processing factor of 0.28 was applied for dietary exposure estimation in this study. Table 7 summarizes the dietary exposure to twelve mycotoxins detected in wheat grain samples for different age subgroups in China. The dietary intake of DON through wheat consumption was in the range of 0.592–0.992 µg/kg b.w./day, not exceeding the PMTDI of 1 µg/kg b.w./day set by JECFA [39], with the highest exposure found in children aged 4–7 years. The HQs of DON for different age groups among the Chinese population were less than 1, indicating a tolerable exposure level for Chinese consumers. Although DON is reported to be stable during processing such as heating, extrusion, cooking, brewing, or baking, physical removal techniques such as sorting, cleaning, and grinding are extremely effective in removing DON from food commodities [40]. As reported by the FAO/WHO JECFA [39], the dietary exposure to DON from wheat consumption was 5.3 µg/kg b.w./day for GEMS/Food Cluster B (Lebanon, United Arab Emirates, Cyprus, Greece, Israel, Italy, Portugal, Spain, and Turkey) and 9.13 µg/kg b.w./day for GEMS/Food Cluster M (the United States and Canada), which are higher than the PMTDI of 1 µg/kg b.w./day [39]. This dietary exposure to DON from wheat consumption ranged from 2.11 to 3.54 µg/kg b.w./day, exceeding the PMTDI value (1 µg/kg b.w./day), when processing factors were not considered, which is consistent with the FAO/WHO JECFA report. As shown in Table 7, the dietary intake of ZEN was 0.007–0.012 µg/kg bw/day, which is lower than the PMTDI of 0.5 µg/kg b.w./day [41], with the corresponding HQs being far lower than 1, implying a tolerable health risk for Chinese consumers. In 2011, the EFSA reported that dietary exposure to ZEN through consumption of grains and grain-based food was acceptable for all age groups [21]. However, it was reported that people of Rajasthan (a state in India) were exposed to three and six tenths to four-fold of TDI of ZEN through wheat consumption [25].

BEA and ENNs are frequently detected in various foodstuffs, and cereal grains and cereal-based food products are the main contributors to their dietary exposure. BEA and ENNs exert cytotoxic effects, thereby affecting the cellular ionic homeostasis [42]. In this study, dietary exposures to BEA and ENNs were evaluated in terms of the TCC value (0.025 µg/kg b.w./day for BEA and 1.5 µg/kg b.w./day for the sum of ENNs) as proposed by the EFSA Panel on Contaminants in the Food Chain (CONTAM) [43] to obtain a rough estimation of the possible health risks to humans. Herein, the estimated levels of dietary exposure to BEA and ENNs were 0.0003–0.0004 μg/kg b.w./day and 0.004–0.007 μg/kg b.w./day, respectively, indicating acceptable health risks for the Chinese population. Similar results were reported in a study conducted in the Romanian population [44]. The dietary exposure to BEA ranged from 0 μg/kg b.w./day (lower-bound scenario, LB) to 0.0053 μg/kg b.w./day (upper-bound scenario, UB), while the dietary exposure to ENNs ranged from 0.0312 μg/kg b.w./day (LB) to 0.0805 μg/kg b.w./day (UB) through the consumption of wheat samples. Although the results indicated that the chronic dietary exposure may not have adverse effects on human health, attention should be paid to the synergistic or additive effects of multi-mycotoxin co-occurrence in wheat grains. Further assessment should be conducted once data on toxicity-guided fractionation of the BEA and ENNs are available in the future.

Owing to the limited toxicological data available for Alternaria toxins, such as AME, AOH, TeA, and TEN, the TTC approach (0.0025 µg/kg b.w./day for AME and AOH; 1.5 µg/kg b.w./day for TeA and TEN) was adopted to assess the effects of these mycotoxins on human health [45]. Regarding AME and AOH, the results of the dietary exposure estimates ranged from 0.003 to 0.007 µg/kg b.w./day, exceeding the TTC value of 0.0025 µg/kg b.w./day, with the corresponding HQs for AME and AOH being >1. Overall, the risk characterization results emphasized that special attention must be paid to AME and AOH contamination in wheat and additional toxicity data and occurrence data must be acquired for a detailed and accurate assessment of health risks. For TeA, the dietary exposure was estimated to be 0.223–0.373 µg/kg b.w./day (Table 7), which is less than the TTC value of 1.5 µg/kg b.w./day, consistent with the reports published by EFSA suggesting that TeA is unlikely to be of human health concern [21]. Compared with AME, AOH, and TeA, TEN has been relatively understudied in food commodities [28]. In this study, the dietary exposure to TEN was in the range of 0.025 to 0.041 µg/kg b.w./day, which was lower than the TCC value of 1.5 µg/kg b.w./day, and the corresponding HQs were <1, indicating tolerable health risk for the Chinese population. The CONTAM Panel of EFSA also reported that TEN is unlikely to have adverse effects on human health [21]. The dietary exposure assessment was not performed for ALT due to the lack of health reference values.

Notably, some uncertainties are involved in the dietary exposure assessment for mycotoxins among Chinese consumers. The dietary risk assessment conducted in this study was only a preliminary analysis due to limited data on the toxicological properties of the emerging BEA, ENNs, and Alternaria toxins. The present study did not evaluate the total dietary exposure but only determined the intake of mycotoxins from wheat grains. Generally, consumers are usually exposed to various chemical hazards with multi-exposure pathways in daily life, and therefore, the possible synergistic or additive effects of multi-mycotoxins or other toxic chemicals (e.g., pesticides or heavy metal) should not be ignored.

## 3. Conclusions

Twelve types of mycotoxins (regulated and emerging mycotoxins) were analyzed by LC–MS/MS in wheat grains randomly collected from eight provinces in China in 2021. The results showed that each wheat grain sample was contaminated with various mycotoxins, and the detection rates of each type of mycotoxin were in the range of 7.1–100%, with the average occurrence level ranging from 1.11 to 921.8 µg/kg. DON and TeA were the predominant mycotoxins with respect to both prevalence and concentration. Furthermore, this study provided further evidence for the co-occurrence of natural multi-mycotoxins in wheat grains. The dietary exposure assessment results showed that the dietary risks of DON, ZEN, BEA, ENNs, TeA, and TEN for Chinese consumers aged 4–70 years were acceptable, while AME and AOH demonstrated potential unacceptable health risks for Chinese consumers. To the best of our knowledge, this study is the first to estimate the dietary health risk of emerging mycotoxins BEA, ENNs, and Alternaria toxins through the assessment of contamination levels in wheat grains in China. Future studies are necessary to reduce the uncertainties associated with toxicities of emerging mycotoxins, processing factors, and cumulative risk assessment of regulated and emerging mycotoxins.

## 4. Material and Methods

### 4.1. Chemicals and Reagents

Reagents of high-performance liquid chromatography (HPLC) grade, including acetonitrile, methanol, formic acid (purity ≥ 99%), and ammonium acetate (purity ≥ 99%) were purchased from Fisher Scientific (Leicestershire, UK). C18 (40–60 µm) was obtained from Shandong Meizheng Bio-Tech Co., Ltd. (Rizhao, China). Purified water was obtained using the Millipore Milli-Q Apparatus (Massachusetts, USA).

The standards of DON, ZEN, BEA, ENA, ENA1, ENB, ENB1, AME, AOH, TEN, TeA, and ALT were obtained from Pribolab (Singapore) Pte. Ltd. (purity ≥ 99.0%. The isotope internal standard (IS) solutions of ^13^C_15_-DON (25 μg/mL) and ^13^C_18_-ZEN (25 μg/mL) were purchased from Romer Labs Inc. (Tullin, Austria). The ^13^C-IS stock solutions of ^13^C_45_-BEA (25.13 μg/mL), ^13^C_36_-ENA (10.01 μg/mL), ^13^C_35_-ENA1 (10.01 μg/mL), ^13^C_33_-ENB (10.01 μg/mL), ^13^C_34_-ENB1 (10.02 μg/mL), ^13^C_15_-AME (25.76 μg/mL), ^13^C_14_-AOH (25.76 μg/mL), ^13^C_22_-TEN (10.0 μg/mL), ^13^C_10_-TeA (25.01 μg/mL), and ^13^C_15_-ALT (10.0 µg/mL) were obtained from Pribolab (Singapore) Pte. Ltd. (Qingdao, China). All analytical standards were kept in the dark at −18 °C during the analysis process. The solvent calibration standards were prepared using a series of dilutions of the standards (100 μg/mL) with methanol/water (50/50, *v*/*v*). The ^13^C-IS spiking solution was fortified into the mixed solvent calibration standard before the LC–MS analysis.

### 4.2. Sample Collection

**Figure 4:**
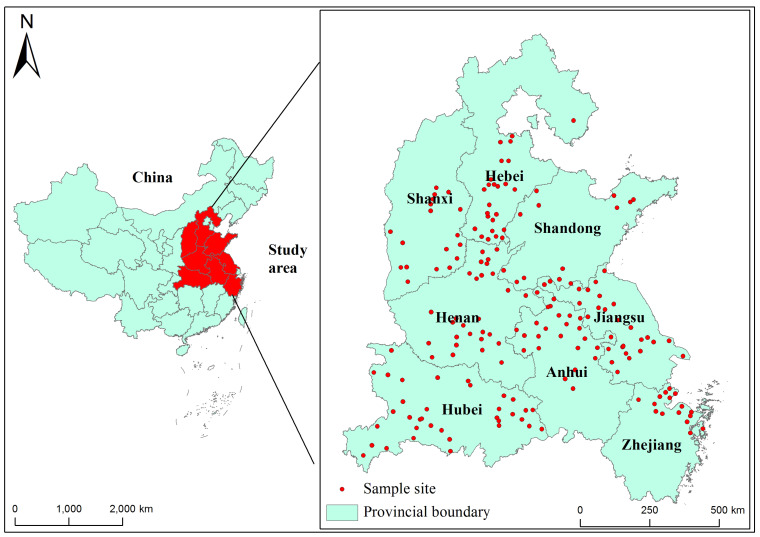
Wheat grain samples collected from the eight main production areas across China.

A total of 321 wheat grain samples were randomly collected from 184 agricultural lands in eight provinces of China, namely Zhejiang (40), Jiangsu (57), Anhui (49), Hubei (36), Henan (30), Shandong (46), Shanxi (31) and Hebei (32), between May 2021 and June 2021 (Figure 4). All samples were collected in accordance with the national standards of GB 5490-2010 [46]. Each sample was pooled from 5 subsamples (500 g for each) supplied by 5 family farms from each sampling point. The collected wheat grain samples were sealed in plastic bags, transferred to the laboratory immediately after collection, and kept in a dry place (approximately 20 °C) with good ventilation before analysis.

In this study, each wheat grain sample was thoroughly ground to a fine powder using a grinding mill (Yongkang Hongsun Electromechanical Co., Yongkang, China), passed through a 2-mm sieve, and mixed thoroughly. Quartering method of sampling [47] was employed in the laboratory. The samples were carefully cleaned to avoid cross contamination between each sample preparation. The analytical samples were stored in plastic bags at −18 °C before LC–MS analysis.

### 4.3. Analytical Method

A modified sample pretreatment method was developed based on a previous study [19] in our laboratory. Briefly, homogenized and representative portions of the samples weighing 5 g were added to 50 mL polypropylene centrifuge tubes, followed by the addition of isotopically labeled internal standards (0.5 mL of ^13^C_15_-DON and ^13^C_45_-TeA mixture (1 μg/mL); 0.1 mL of ^13^C_18_-ZEN and ^13^C_22_-TEN mixture (1.0 μg/mL); 0.05 mL of ^13^C_45_-BEA, ^13^C_36_-ENA, ^13^C_35_-ENA1, ^13^C_33_-ENB, ^13^C_34_-ENB1, ^13^C_15_-AME, ^13^C_14_-AOH, and ^13^C_10_-ALT mixture (1 μg/mL)). The mycotoxins were extracted by adding 20 mL of acetonitrile/water/formic acid (79:20:1, *v*/*v*/*v*) solution. The tubes were then subjected to sonication for 30 min at 25 °C by using an XM-P102H ultrasonic cleaner 40KHz (Kunshan, China), shaken for 5 min using a multi-tube vortex mixer Mix200 (Shanghai, China), allowed to stand for 20 min, and then shaken again for 5 min on the multi-tube vortex mixer. Then, the tubes were centrifuged at 9693× *g* for 5 min at 25 °C by using the Thermo Scientific Sorvall ST8R centrifuge (Waltham, MA, USA). Next, 6 mL of the supernatants was cleaned with 200 mg C18. The tubes were vigorously shaken for 30 s and centrifuged at 9693× *g* for 5 min at 25 °C. Then, 4 mL of the supernatants was allowed to evaporate to dryness at 40 °C under a gentle stream of nitrogen by using the AutoEVA-60 Automatic Parallel Concentrator (Xiamen, China). The dried extract was reconstituted in 1 mL of methanol/water solution (1:1, *v*/*v*) and vortexed for approximately 30 s. The extract was filtered (0.22 μm) and analyzed by LC–MS/MS.

### 4.4. LC–MS/MS Analysis

Chromatographic separation of the mycotoxins was performed with LC–MS/MS. The HPLC was equipped with a Shimadzu LC-30AD quaternary pump (Shimadzu, Tokyo, Japan), SIL-30AC autosampler, and CTO-20AC column oven. The Waters ACQUITY BEH C18 analytical column (2.1 × 100 mm, 1.7 μm) protected by the Waters ACQUITY BEH C18 guard cartridge (2.1 × 5 mm, 1.7 μm) served as the stationary phase. The column temperature was maintained at 40 °C, the flow rate was set to 0.2 mL/min, and the injection volume was 5 µL. The mobile phase comprised 5 mmol/L ammonium acetate (phase A) and methanol (B). The gradient was set as follows: 0–1.0 min, 5% B; 1.0–6.0 min, 5–95% B; 4.0–8.0 min, 95% B; 8.0–8.1 min, 95–5% B; 8.1–10 min, 5% B. The total run time was 10 min. The MS/MS was AB 5500 triple quadrupole mass spectrometer (AB Sciex, Framingham, MA, USA) equipped with a Turbo-V^TM^ electrospray ionization source (ESI) interface. Multiple reaction monitoring (MRM) scanning was used for quantification, and the electrospray ion source polarity between positive and negative modes was switched in a single chromatographic run.

The applied parameters were as follows: ion spray voltage, 5500 V for the positive ion mode and −4500 V for the negative ion mode; source temperature, 500 °C; curtain gas, 35 psi; collision gas, 8 psi; ion source gas 1 (GS1), 50 psi; and ion source gas 2 (GS2), 55 psi. The LC–MS/MS acquisition parameters for the 12 mycotoxins are listed in Table S1.

### 4.5. Quality Control

The calibration curves for individual mycotoxin by solvent spiked with the standards and internal standards were prepared before the quantification of the individual mycotoxin in wheat grains. The stability and reproducibility of the instruments were monitored by analyzing two levels of reference standards at the beginning and the end of each measurement. Duplicates were measured and the means were calculated.

### 4.6. Risk Assessment

The assessments of dietary exposure to 12 mycotoxins were conducted using a deterministic approach. The estimated daily intake (EDI) of mycotoxins was calculated as follows: EDI (µg/kg b.w./day) = (average concentration of mycotoxins in wheat samples [µg/kg] × consumption of wheat [kg/day]) × processing factor/body weight (kg). In this study, the dietary exposure assessment was estimated based on the mean level of relevant mycotoxins detected in wheat grains, average consumption of wheat grains, wheat processing factor, and body weight of Chinese residents aged 4–70 years. Information on estimated wheat consumption and body weight of the different Chinese subgroups was obtained using data from “Survey Report on Nutrition and Health Status of Chinese Residents Part II—Diet and Nutrition Intake Status 2002” [48] and “Survey Report on Nutrition and Health Status of Chinese Residents Part III—Physical and Nutrition Status of Chinese Residents 2002” [49]. During the processing of wheat into flour, the processing factors for various mycotoxins ranged from 0.35 to 0.21, which was obtained from our unpublished data. In this study, the average processing factor of 0.28 was used for dietary exposure estimation.

Risk characterization is expressed as the hazard quotient (HQ), which was estimated as the ratio of outputs of the dietary exposure to the health-based guidance values (HBGVs) for each mycotoxin. The HQ was calculated using the following formula: HQ = EDI/HBGV, with the HQ < 1 indicating a tolerable exposure level and the HQ > 1 indicating a non-tolerable exposure level [50].

The Joint FAO/WHO expert Committee on Food Additives (JECFA) has established a provisional maximum tolerable daily intake (PMTDI) of 1 μg/kg b.w./day for DON and its acetylated derivatives, which commonly serves as the basis for assessing chronic dietary exposure [39]. The PMTDI for ZEN has been set to 0.5 μg/kg b.w./d by the JECFA [35]. However, for BEA and ENNs (the sum of ENA, ENA1, ENB, and ENB1), no reference dose (e.g., PMTDI) is available owing to the lack of toxicity data in vivo. To gain insights into the possible risks of dietary exposure to BEA and ENNs, the EFSA Panel on Contaminants in the Food Chain (CONTAM) proposed the estimated chronic dietary exposure levels compared with the Threshold of Toxicological Concern (TTC) value (0.025 μg/kg b.w./day for BEA and 1.5 μg/kg b.w./day for the sum of ENNs) [42]. Considering the limited toxicity data available for Alternaria toxins, the dietary exposure to AME, AOH, TeA, and TEN was estimated based on TTC value (0.0025 μg/kg b.w./day for AME and AOH; 1.5 μg/kg b.w./day for TeA and TEN) [51,52]. To date, PMTDI or TTC value for ALT is lacking.

### 4.7. Statistical Analysis

The samples with mycotoxin content more than the LOQ were considered positive, whereas those with mycotoxin content less than the LOQ were considered negative. In the evaluation of the occurrence of regulated and emerging mycotoxins in the wheat grain samples, non-detection data were treated as “0”, and non-detection data were treated as half the LOD while estimating the dietary exposure [17]. MultiQuant 3.0 (Sciex, Framingham, MA, USA) was used for chromatographic data analysis and processing.

Correlations between each two mycotoxins were analyzed through Pearson’s test in the Scipy package Pycharm (2022.1.3, JetBrains). *t*-test was performed to compare two independent samples and Kruskal–Wallis one-way analysis of variance was applied to compare more than three independent variables. Pearson’s test was applied to assess the linear correlation between two variables. The statistically significant level (*p*-value) was set to 0.05.

## Figures and Tables

**Table 1:** Recoveries and relative standard deviations (*n* = 6) of twelve mycotoxins in the wheat grain samples.

Analyte	Spiked Level (μg/kg)	Average Recovery (%)	Relative Standard Deviation (RSD) (%)
DON	100	101	5.76
	1000	101	3.77
	2000	103	4.11
ZEN	20	110	7.28
	50	98.2	6.34
	100	92.3	3.46
BEA	1	89.6	5.31
	5	97.2	3.56
	10	95.6	8.31
ENA	1	92.5	11.2
	5	94.6	7.23
	10	97.4	5.72
ENA1	5	89.2	5.19
	10	87.9	10.8
	50	90.8	4.60
ENB	5	91.6	3.40
	10	96.3	5.26
	50	97.5	3.32
ENB1	1	98.9	7.64
	5	101	10.2
	10	97.2	10.5
AME	1	89.5	6.11
	5	91.7	4.95
	10	89.9	3.43
AOH	5	107	5.87
	10	98.5	3.81
	50	96.7	4.19
TeA	50	98.2	11.9
	100	94.5	3.27
	200	93.2	5.54
TEN	1	107	10.1
	5	101	3.52
	10	103	6.35
ALT	10	112	8.41
	50	97.8	5.16
	100	96.7	11.1

**Table 2:** The limit of detection (LOD) and limit of quantification (LOQ) of twelve mycotoxins in the wheat grain samples.

Analytes	Correlation Coefficient (*R*)	Limit of Detection in Matrix, LOD (μg/kg)	Limit of Quantification in Matrix, LOQ (μg/kg)
DON	0.9996	3.0	10.0
ZEN	0.9978	0.3	1.0
BEA	0.9982	0.2	0.6
ENA	0.9984	0.1	0.3
ENA1	0.9920	0.5	1.5
ENB	0.9997	0.03	0.1
ENB1	0.9936	0.3	1.0
AME	0.9985	0.3	1.0
AOH	0.9996	0.5	1.5
TeA	0.9977	1.0	3.0
TEN	0.9995	0.3	1.0
ALT	0.9981	0.3	1.0

**Table 3:** The detection frequency and concentration of twelve mycotoxins in the wheat grain samples collected from China.

Analytes	Positive/Total	Detection Frequency (%)	Mean ± SD (μg/kg)	Median (μg/kg)	Maximum (μg/kg)
DON	307/321	95.6	921.8 ± 900	626.9	8116
ZEN	172/321	53.6	19.7 ± 40.0	6.70	220
BEA	38/321	11.8	2.44 ± 4.38	1.38	27.0
ENA	108/321	33.6	1.11 ± 1.50	0.566	9.26
ENA1	117/321	36.4	9.87 ± 12.3	5.50	68.2
ENB	243/321	75.7	10.8 ± 21.1	2.24	136
ENB1	177/321	55.1	19.9 ± 30.7	8.63	190
AME	215/321	67.0	4.89 ± 7.34	2.81	71.6
AOH	200/321	62.3	8.72 ± 10.1	5.07	102
TeA	321/321	100	331.9 ± 288.8	258.9	2034
TEN	319/321	99.4	37.0 ± 26.5	31.5	133
ALT	23/321	7.17	2.05 ± 1.53	1.37	6.17

Note. Detection results < LOQ is considered as “non-detect”, and the input is “0” during mathematical statistical analysis [17].

**Table 4:** The frequency distribution of the levels of DON, ZEN, and TeA in wheat grain samples (*n* = 321).

Mycotoxin	Range of Concentration (μg/kg)	Number of Samples	Frequency (%)
DON	>3000	1	0.31
	2000–3000	34	10.6
	1000–2000	65	20.2
	<1000	221	68.8
ZEN	>200	2	0.62
	100–200	8	2.49
	60–100	1	0.31
	<60	310	96.6
TeA	1000–2000	11	3.43
	500–1000	38	11.8
	200–500	166	51.7
	<500	106	33.0

**Table 5:** The investigation outcomes of twelve mycotoxins in wheat grain samples collected from the main producing areas in China.

Location	Statistics	DON	ZEN	BEA	ENA	ENA1	ENB	ENB1	AME	AOH	TeA	TEN	ALT
Zhejiang	Detection frequency (%)	100	75.0	47.5	5.00	5.00	27.5	20.0	52.5	37.5	100	100	2.50
	5th	258	1.37	0.71	–	–	–	–	1.02	–	52.5	1.38	–
	25th	337	2.45	0.99	–	–	0.31	1.13	1.40	9.79	98.4	2.37	–
	Mean	614	11.8	1.42	0.23	1.62	1.18	2.81	5.93	17.67	227	9.09	1.08
	SD	264	13.40	0.48	0.02	0.05	1.15	2.17	4.86	10.25	155	9.20	–
	Median	590	6.70	1.36	0.23	1.62	0.63	1.46	5.27	13.31	214	7.22	1.08
	75th	756	17.4	1.84	–	–	2.46	5.08	7.93	27.7	339	11.1	–
	95th	1074	50.4	–	–	–	–	–	17.1	–	524	29.6	–
Jiangsu	Detection frequency (%)	100	71.9	7.02	17.5	22.8	80.7	43.9	71.9	75.4	100	100	3.51
	5th	193	1.03	–	–	–	0.120	–	1.17	1.60	62.8	8.39	–
	25th	412	3.88	–	0.29	2.33	0.198	1.47	1.81	2.85	182	17.6	–
	Mean	1150	10.3	1.07	0.57	5.01	2.62	6.46	3.53	9.35	244	24.9	–
	SD	1292	8.43	0.31	0.30	3.46	5.46	7.43	2.12	7.71	119	10.2	–
	Median	1001	11.1	0.90	0.46	3.39	0.67	3.72	3.09	8.75	222	24.5	–
	75th	1439	17.0	–	0.85	6.84	2.07	10.1	6.13	18.7	256	29.9	–
	95th	1892	30.2	–	–	–	19.9	–	8.09	24.8	407	37.8	–
Anhui	Detection frequency (%)	100	69.4	16.3	36.7	36.7	89.8	61.2	53.1	63.3	100	100	8.16
	5th	167	1.69	–	–	–	0.136	1.23	1.43	1.60	45.7	4.69	–
	25th	733	4.20	0.736	0.237	1.81	0.441	2.49	2.12	5.26	132	17.4	–
	Mean	1649	59.7	2.43	0.532	4.84	4.24	9.91	3.93	9.72	254	24.1	2.24
	SD	1054	79.6	1.58	0.300	2.91	4.67	8.81	2.91	8.51	219	11.4	1.10
	Median	1521	12.7	2.95	0.533	4.50	2.26	5.77	3.02	9.08	235	25.2	2.05
	75th	2708	143	3.67	0.847	7.67	7.98	16.4	4.57	12.38	283	32.0	3.02
	95th	3157	215	–	–	–	14.1	29.3	11.8	36.7	1133	42.9	–
Hubei	Detection frequency (%)	100	38.9	2.78	75.0	86.1	91.7	100	97.2	100	100	100	2.78
	5th	26.5	–	–	0.236	1.80	2.57	3.07	1.66	2.66	155	48.3	–
	25th	167	1.78	–	0.443	4.07	9.50	11.24	2.13	3.46	337	64.0	–
	Mean	819	7.29	1.44	1.16	11.6	35.5	33.2	3.47	5.15	434	80.1	2.47
	SD	808	6.43	–	1.00	10.9	36.6	30.7	2.91	3.32	155	20.1	–
	Median	631	6.15	1.44	0.85	9.54	20.7	17.2	2.44	4.37	433	73.3	2.47
	75th	1602	10.7	–	1.73	14.7	61.5	54.4	4.02	5.57	547	101	–
	95th	2639	–	–	4.39	46.6	130.2	118.5	13.3	16.1	776	112	–
Henan	Detection frequency (%)	100	40.0	10.0	70.0	86.7	96.7	90.0	76.7	90.0	100	100	10.0
	5th	37.0	–	–	0.27	1.89	0.77	3.36	1.25	1.54	107	16.9	–
	25th	533	1.54	1.02	0.53	4.76	8.38	12.8	1.61	2.03	187	44.9	1.25
	Mean	926	12.2	1.85	1.97	17.1	28.1	47.4	7.18	6.90	350	58.4	3.73
	SD	553	13.5	1.12	2.28	18.5	25.9	50.4	11.48	7.12	352	24.6	2.82
	Median	779	8.10	1.41	1.07	11.0	21.5	30.3	3.07	3.32	280	58.1	3.77
	75th	1264	17.58	3.13	3.68	31.6	47.6	85.4	6.89	9.36	383	72.8	6.17
	95th	1977	–	–	–	67.9	90.9	187.1	51.6	23.0	1637	122	–
Shandong	Detection frequency (%)	100	30.43	4.35	17.4	19.6	67.4	37.0	67.4	69.6	100	100	2.17
	5th	43.1	–	–	–	–	0.13	–	1.01	1.62	57.7	9.68	–
	25th	162	1.20	–	0.28	2.83	0.24	2.10	1.20	1.82	223	28.1	–
	Mean	633	3.15	13.9	1.58	6.90	2.78	8.86	3.34	3.48	537	42.3	1.00
	SD	673	2.42	18.5	2.30	6.20	5.86	13.44	7.07	3.24	453	24.3	–
	Median	391	2.00	13.9	0.57	3.31	0.44	4.48	1.45	2.58	325	35.9	1.00
	75th	1131	4.05	–	1.74	11.6	3.00	11.4	2.42	4.32	865	64.4	–
	95th	1830	–	–	–	–	22.2	–	27.8	13.2	1684	97.7	–
Shanxi	Detection frequency (%)	58.1	12.9	3.23	22.6	12.9	54.8	25.8	25.8	19.4	100	93.5	29.0
	5th	–	–	–	–	–	–	–	–	–	4.10	6.24	–
	25th	15.3	3.20	–	0.21	1.92	0.26	1.23	1.21	2.31	42.8	12.7	1.14
	Mean	651	5.88	9.57	0.70	4.55	1.72	7.84	8.35	25.9	232	25.8	1.64
	SD	1227	3.29	–	0.75	4.28	3.35	13.0	11.8	40.4	328	17.9	1.07
	Median	26.5	4.20	9.57	0.36	2.71	0.42	2.49	2.43	22.3	144	20.2	1.20
	75th	82.8	10.60	–	1.67	9.02	1.99	9.74	19.8	87.0	198	35.1	1.63
	95th	–	–	–	–	–	–	–	–	–	1347	72.5	–
Hebei	Detection frequency (%)	100	71.9	–	46.9	43.8	100	81.3	93.8	84.4	100	100	3.13
	5th	26.0	1.42	–	–	–	0.110	1.29	1.25	1.67	93.6	35.1	–
	25th	304	3.55	–	0.23	2.13	0.67	1.95	2.47	3.83	348	41.6	–
	Mean	449	7.51	–	1.03	8.83	6.75	14.6	6.69	6.32	401	47.7	1.07
	SD	245	5.68	–	1.78	12.9	12.0	27.2	12.6	3.3	174	11.8	–
	Median	464	5.20	–	0.45	4.27	2.53	4.85	4.00	5.35	376	46.5	1.07
	75th	555	6.70	–	1.28	10.9	8.74	16.1	5.87	7.69	439	50.0	–
	95th	904	22.50	–	–	–	47.7	123	54.7	14.8	723	85.2	–

Note. Detection results (<LOQ) is considered as “non-detect”, and the input is “0” during mathematical statistical analysis.

**Table 6:** A summary of the combinations of co-occurrence of regulated and emerging mycotoxins detected in wheat grain samples.

N	Co-Occurrence	Frequency (%)	N	Co-Occurrence	Frequency (%)
1 toxin	TeA	0.312	7 toxins	DON + ZEN + ENB + AME + AOH + TeA + TEN	7.17
2 toxins	TeA + TEN	0.623		DON + ENA + ENA1 + ENB + ENB1 + TeA + TEN	2.80
	ENB + TeA	0.312		DON + ENB + ENB1 + AME + AOH + TeA + TEN	2.49
3 toxins	DON + TeA + TEN	4.05		DON + ZEN + BEA + AME + AOH + TeA + TEN	1.56
	ENB + TeA + TEN	0.623		others	3.43
	TeA + TEN + ALT	0.623	8 toxins	DON + ZEN + ENB + ENB1 + AME + AOH + TeA + TEN	7.48
4 toxins	DON + ENB + TeA + TEN	4.36		DON + ENA1 + ENB + ENB1 + AME + AOH + TeA + TEN	1.87
	DON + ZEN + TeA + TEN	2.18		DON + ZEN + ENA + ENA1 + ENB + ENB1 + TeA + TEN	1.25
	DON + BEA + TeA + TEN	1.25		DON + ENA + ENB + ENB1 + AME + AOH + TeA + TEN	0.93
	DON + AOH + TeA + TEN	0.935		others	4.05
	others	1.56	9 toxins	DON + ENA + ENA1 + ENB + ENB1 + AME + AOH + TeA + TEN	8.41
5 toxins	DON + AME + AOH + TeA + TEN	3.12		DON + ZEN + ENA1 + ENB + ENB1 + AME + AOH + TeA + TEN	3.12
	DON + ZEN + BEA + TeA + TEN	1.25		DON + ZEN + ENA + ENB + ENB1 + AME + AOH + TeA + TEN	1.25
	DON + ZEN + AME + TeA + TEN	1.25		DON + ZEN + BEA + ENB + ENB1 + AME + AOH + TeA + TEN	0.935
	DON + ZEN + ENB + TeA + TEN	1.25		others	1.87
	others	3.74	10 toxins	DON + ZEN + ENA + ENA1 + ENB + ENB1 + AME + AOH + TeA + TEN	10.3
6 toxins	DON + ZEN + AME + AOH + TeA + TEN	2.49		DON + ENA + ENA1 + ENB + ENB1 + AME + AOH + TeA + TEN + ALT	0.623
	DON + ENB + AME + AOH + TeA + TEN	2.18		DON + BEA + ENA + ENA1 + ENB + ENB1 + AME + AOH + TeA + TEN	0.623
	DON + ZEN + ENB + ENB1 + TeA + TEN	0.935		DON + ZEN + BEA + ENA1 + ENB + ENB1 + AME + AOH + TeA + TEN	0.312
	DON + BEA + ENB + ENB1 + TeA + TEN	0.623	11 toxins	DON + ZEN + BEA + ENA + ENA1 + ENB + ENB1 + AME + AOH + TeA + TEN	1.25
	DON + ZEN + ENB + AOH + TeA + TEN	0.623		DON + ZEN + ENA + ENA1 + ENB + ENB1 + AME + AOH + TeA + TEN + ALT	0.935
	others	2.80	12 toxins	DON + ZEN + BEA + ENA + ENA1 + ENB + ENB1 + AME + AOH + TeA + TEN + ALT	0.312

**Table 7:** Dietary exposure to twelve mycotoxins detected in wheat grain samples for different age subgroups in China.

Age Groups (Years)	Dietary Exposure (EDI, μg/kg b.w./day)								Risk Characterization (HQ, %)							
	DON	ZEN	BEA	ENNs	AME	AOH	TeA	TEN	DON	ZEN	BEA	ENNs	AME	AOH	TeA	TEN
4–7	0.992	0.012	0.0004	0.007	0.004	0.007	0.373	0.041	99.2	2.39	1.69	0.44	149.8	269.1	24.9	2.76
7–11	0.832	0.01	0.0004	0.006	0.004	0.006	0.313	0.035	83.2	2.01	1.42	0.37	125.7	225.7	20.9	2.31
11–14	0.74	0.009	0.0003	0.005	0.003	0.005	0.279	0.031	74.0	1.78	1.26	0.33	111.8	200.8	18.6	2.06
14–18	0.685	0.008	0.0003	0.005	0.003	0.005	0.258	0.029	68.5	1.65	1.17	0.3	103.4	185.7	17.2	1.9
18–30	0.651	0.008	0.0003	0.004	0.003	0.004	0.245	0.027	65.1	1.57	1.11	0.29	98.4	176.7	16.3	1.81
30–45	0.62	0.007	0.0003	0.004	0.003	0.004	0.233	0.026	62	1.49	1.06	0.27	93.6	168.2	15.6	1.72
45–60	0.607	0.007	0.0003	0.004	0.003	0.004	0.229	0.025	60.7	1.46	1.04	0.27	91.7	164.7	15.2	1.69
60–70	0.592	0.007	0.0003	0.004	0.003	0.004	0.223	0.025	59.2	1.43	1.01	0.26	89.4	160.6	14.9	1.64

Note. EDI = (mean concentration of mycotoxins detected in wheat grains × consumption of wheat grains) × processing factor/body weight, μg/kg b.w./day. During data treatment, LOD/2 values were used for the data below LOD. Mean concentrations of DON, ZEN, BEN, ENNs, AME, AOH, TeA, TEN, and ALT were 881.7, 10.6, 0.377, 5.85, 3.33, 5.98, 331.9, 36.8, and 0.286 μg/kg in wheat grain samples, respectively; processing factor = 0.28. HQ = (EDI/TDI or TTC) × 100%. Health risk is considered acceptable when the HQ is lower than 100%; health risk is considered unacceptable when the HQ is more than 100%. Tolerable daily intake (TDI) for DON and ZEN is 1.0 and 0.5 μg/kg b.w./day, respectively. Threshold of toxicological concern (TTC) for BEA and ENNs is 0.025 and 1.5 μg/kg b.w./day, respectively; TTC for AME and AOH is 0.0025 μg/kg b.w./day; and TTC for TeA and TEN is 1.5 μg/kg b.w./day, respectively.

## Data Availability

The data presented in this study are available in this article and supplementary material.

## Supplementary Materials

The following supporting information can be downloaded at: https://www.mdpi.com/article/10.3390/toxins15060389/s1, Figure S1: Shows the LC–MS/MS chromatogram of mycotoxins DON, ZEA, BEA, ENA, ENA1, ENB, ENB1, AME, AOH, TeA, TEN, and ALT in the wheat grain matrix. The concentration level of DON is 50 μg/kg; the concentration level for both of TeA and ALT is 10 μg/kg; the remaining mycotoxins are at 5 μg/kg.; Table S1: The LC-MS/MS acquisition parameters for the twelve mycotoxins.
