# Supplementary material for: Natural Occurrence of Regulated and Emerging Mycotoxins in Wheat Grains and Assessment of the Risks from Dietary Mycotoxins Exposure in China
Source: Toxins (Basel). 2023 Jun 9;15(6):389. doi: 10.3390/toxins15060389 (PMC10300805; doi:10.3390/toxins15060389)
Supplement: Supplementary file 1 [file toxins-15-00389-s001.zip › toxins-2408747-supplementary.pdf]

toxins

Supplementary Materials

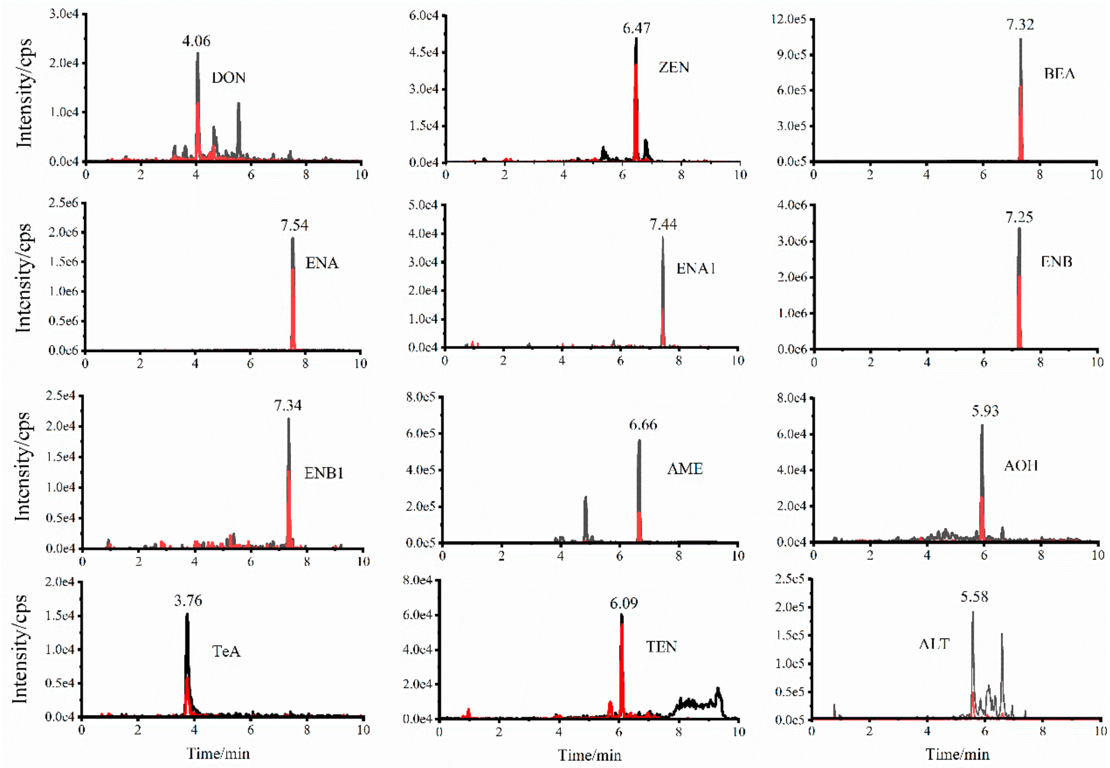

Figure S1. The LC–MS/MS chromatogram of mycotoxins DON, ZEA, BEA, ENA, ENA1, ENB, ENB1, AME, AOH, TeA, TEN, and ALT in the wheat grain matrix. The concentration level of DON is 50μg/kg; the concentration level for both of TeA and ALT is 10 μg/kg; the remaining mycotoxins are at 5 μg/kg.

Table S1. The LC-MS/MS acquisition parameters for the 12 mycotoxins.

Mycotoxin	Precursor ion (m/z)	Product ions (m/z)	Declustering potential (V)	Collision energy (eV)
DON	295.1	265.1*	-32	-15
		138.1	-32	-23
ZEN	317.2	175.0*	-153	-32
		131.0	-153	-39
BEA	801.3	784.5*	50	23
		243.9	50	39
ENA	699.4	682.3*	40	24
		210.1	40	38
ENA1	668.6	210.2*	125	29
		541.2	125	30
ENB	657.4	640.3*	40	24
		196.1	40	40
ENB1	654.7	196.2*	100	36
		210.2	100	30
AME	271.2	256.2*	-100	-28

		228.2	-100	-42
AOH	257.0	213.2*	-120	-32
		147.2	-120	-42
TeA	196.0	139.0*	-50	-28
		112.0	-50	-40
TEN	413.3	140.8*	-126	-27
		271.1	-126	-23
ALT	293.0	257.0*	57	19
		229.0	57	30
$^{13}C_{15}$-DON	310.1	278.9	-31	-16
$^{13}C_{18}$-ZEN	334.9	140.2	-78	-38
$^{13}C_{45}$-BEA	846.5	829.6	80	29
$^{13}C_{36}$-ENA	735.7	718.6	96	29
$^{13}C_{35}$-ENA1	720.7	703.5	74	26
$^{13}C_{33}$-ENB	690.7	673.5	99	26
$^{13}C_{34}$-ENB1	705.7	688.6	86	27
$^{13}C_{15}$-AME	286.2	270.1	-114	-29
$^{13}C_{14}$-AOH	271.1	227.0	-140	-37
$^{13}C_{10}$-TeA	206.1	145.1	-95	-26
$^{13}C_{22}$-TEN	435.1	147.1	-130	-26
$^{13}C_{15}$-ALT	306	213.7	95	34

* Quantification ion transition.
